# A log-binomial Bayesian geoadditive semiparametric analysis of geographical inequalities in caesarean births in Ghana

**DOI:** 10.1186/s12884-023-06087-2

**Published:** 2023-11-10

**Authors:** Fiifi Amoako Johnson

**Affiliations:** Department of Population and Health, Faculty of Social Sciences, College of Humanities and Legal Studies, University of Cape Coast, Cape Coast, Ghana

**Keywords:** Caesarean section, Caesarean births, Geographical inequalities, Geospatial, Maternal Health Survey, Log-binomial, Bayesian geoadditive semiparametric regression, Ghana

## Abstract

**Background:**

Caesarean section is a clinical intervention aimed to save the lives of women and their newborns. In Ghana, studies have reported inequalities in use among women of different socioeconomic backgrounds. However, geographical differentials at the district level where health interventions are implemented, have not been systematically studied. This study examined geographical inequalities in caesarean births at the district level in Ghana. The study investigated how pregnancy complications and birth risks, access to health care and affluence correlate with geographical inequalities in caesarean section uptake.

**Methods:**

The data for the analysis was derived from the 2017 Ghana Maternal Health Survey. The log-binomial Bayesian Geoadditive Semiparametric regression technique was used to examine the extent of geographical clustering in caesarean births at the district level and their spatial correlates.

**Results:**

In Ghana, 16.0% (95% CI = 15.3, 16.8) of births were via caesarean section. Geospatial analysis revealed a strong spatial dependence in caesarean births, with a clear north-south divide. Low frequencies of caesarean births were observed among districts in the northern part of the country, while those in the south had high frequencies. The predominant factor associated with the spatial differentials was affluence rather than pregnancy complications and birth risk and access to care.

**Conclusions:**

Strong geographical inequalities in caesarean births exist in Ghana. Targeted and locally relevant interventions including health education and policy support are required at the district level to address the overuse and underuse of caesarean sections, to correspond to the World Health Organisation recommended optimal threshold of 10% to 15%.

## Background

Caesarean section is a major operative procedure aimed as an intervention for saving the lives of mothers and their newborns from life-threatening pregnancy and childbirth complications [1, 2]. Nonetheless, research evidence also shows that caesarean section may also lead to adverse short- and long-term health problems for women and children [1, 2]. The World Health Organisation (WHO) recommends that a caesarean section is only essential where it is needed to save the life of the mother and newborn [3]. WHO guidance recommends a national-level caesarean section rate of between 10% to 15% as the required benchmark to improve maternal and perinatal outcomes and prevent maternal and neonatal morbidity and mortality [3].

Statistical evidence shows that, globally, caesarean section rates are increasing rapidly. Multicountry data collected between 2010 and 2018 and covering over 95% of all live births estimated that 21.1% of women gave birth through caesarean section [4, 5]. The lowest rate of 5% was reported in sub-Saharan Africa, compared to 42.8% in Latin America and the Caribbean [4]. In all regions of the world, caesarean section rates increased by more than 30.0% since 1990, except for sub-Saharan Africa (+3.6%) and Northern America (+9.5%) [4]. Caesarean section rates in much of sub-Saharan Africa remain low, with uptake below the WHO recommended rate and with only trivial increases over time [3, 5]. The high and increasing rates of caesarean section uptake in some regions of the world are indicative of its overuse and are often not professionally indicated. Contrarily, in other parts of the world, the rates are below the WHO recommendation on what is needed to impact women’s and newborn lives. Excessive use of caesarean section has been shown to have no medical benefits, does not result in a reduction in maternal and perinatal mortality and may rather cause harm and waste of resources [6–9], whilst low uptake exposes women and their newborns to potential obstetric risk and harm [10]. In many low- and middle-income countries, caesarean sections are inaccessible for the poor who needs it but overutilised by the privileged who do not it [11]. Thus, optimal use of caesarean sections has become a public health challenge and a global concern.

At the national level, there are marked inequalities in caesarean births. Research evidence in sub-Saharan Africa shows significantly higher rates among richer, urban and educated women when compared to their poorer, rural and uneducated counterparts [5, 10, 12]. In Ghana, the 2017 Maternal Health Survey (GMHS) reported a national caesarean section prevalence rate of 16.0%, with nine percent, decided before and seven percent on the onset of labour pains. Geographic and socioeconomic inequalities were very high. At the regional level, caesarean section rates ranged from 6.7% in the Upper East region to 23.6% in the Greater Accra region [13]. Three (Northern, Upper East and Upper West) out of the 10 regions (as demarcated at the time of the 2017 GMHS) had rates below 10.0%, whilst two others (Brong Ahafo and Western) had rates below 15%, the WHO recommended benchmark [13]. Socioeconomically, 10.7% of women with no formal education gave birth through caesarean section, compared to 29.1% of those with more than secondary education and also, only 8.5% of women from the poorest households had caesarean birth compared to 26.3% of those from the richest households [13]. These disparities in caesarean section rates are indicative of significant barriers to accessing the intervention by the poor and marginalised regions and unnecessary and overuse among the rich and in the more developed regions. These statistics show that in Ghana, clinical indication alone does not determine access to caesarean section.

It is worth noting that, in Ghana, disparities in caesarean births at the district level where health policies are implemented and monitored are unknown. National and regional level differentials often mask district-level inequalities. Additionally, factors associated with geographical inequalities at the district level are also unknown. Thus, information to monitor and strengthen both under and over-utilisation of caesarean sections is not available. In this regard, this study examines geographical inequalities in caesarean births by identifying districts with significantly low and those with significantly high uptake and associated factors with both high and low clustering of caesarean section uptake. The objective was to investigate whether pregnancy complications and birth risks are the predominant correlates of geographical differentials in caesarean section uptake, compared to access to healthcare care and affluence. All things being equal, it would be expected that pregnancy complications and birth risks would be the main motivating factor for seeking caesarean birth. The study has the potential to inform targeted interventions for addressing both the overuse and underuse of caesarean sections in Ghana.

## Methods

### Data

The data for the analysis was derived from the 2017 Ghana Maternal Health Survey (GMHS) [14]. The 2017 GMHS adopted a two-stage stratified (by region and urban-rural areas) cluster sampling design, using the 2010 Ghana Population and Housing Census as the sampling frame to select Primary Sampling Units (PSUs) and households. A total of 900 (466 urban and 434 rural) PSUs (census enumeration areas) were selected, with 30 households selected from each PSU, resulting in a total sample of 27,000 households. Within the selected households, 25,062 women aged 15–49 years were interviewed. This study covered 8991 women who had a birth 5 years preceding the survey and for whom complete data were available. The 2017 GMHS collected detailed demographic and health information from respondents including births in the last 5 years. The survey also collected information on births by caesarean section for women aged 15-49 years. The dependent variable for the analysis was binary coded 1 if a woman aged between 15 and 49 years who had a birth in the 5 years preceding the survey had a caesarean birth and 0 otherwise. The independent variables for the analysis were selected based on the literature [9, 10, 15–18] and their availability in the GMHS data. They were grouped into three categories –(1) pregnancy complications and birth risks, (2) access to health care, and (3) socioeconomic factors. The 2017 GMHS asked respondents if they suffered from any problem at any time just before, during or after the birth (pregnancy complications) of their last baby. Those who reported suffering from any problem were further asked about the problem they suffered. Following previous studies [19, 20], the reported complications were grouped into six categories (Table 1).

**Table 1:** Pregnancy complications reported by women before, during or after delivery

**Reported complication**	**Type of complication**
Headaches	Pregnancy induced hypertension
Blurred vision	
High fever	
Oedema/pre-eclampsia	
Conclusion/eclampsia	
Excessive bleeding	Haemorrhage
Prolonged labour	Prolonged/obstructed labour
Obstructed labour	
Low baby movement	Vaginal/placental
Hands/feet of baby came out first	
Torn uterus	
Previa/retained placenta	
Fistula	
Foul smelly discharge	
Abdominal pain	Abdominal pain
Other complication	Other complication

The birth risk covariates were the birth outcome categorised as born alive, born dead, miscarriage or abortion and the birth weight of the child was categorised as don’t know/missing, less than 2.5 kg, 2.5 - 3.99 kg and 4.5 kg or higher. The covariates used to measure access to health care included health insurance coverage categorised into covered, not covered (registered but coverage expired) and not registered, the timing of first antenatal care grouped into the first trimester, second trimester and after the second trimester or no antenatal care and place of delivery (public or private facility). The access to health care covariates also included problems getting permission to seek care, problems getting money for treatment, distance or no nearby health facility and not wanting to go alone, all categorised as a big problem or not a big problem. The socioeconomic covariates were the type of place of residence (urban and rural), marital status (currently married, cohabiting and not in a union), educational attainment (no education, primary, middle school/Junior High School, Senior High School and higher), religious affiliation (Catholic, Protestant, Pentecostal/Charismatic, Other Christians, slam and no religion/Traditionalist/Spiritualist), and ethnicity (Akan, Ga-Dangbe, Ewe-Guan, Mole-Dagbane, Grussi-Gruma-Mande and Other). The other socioeconomic covariates were household wealth status (poorest, poorer, middle, rich and richest), reading newspaper (at least once a week, less than once a week, not at all and cannot read), listening to radio and watching television, categorised as at least once a week, less than once a week and not at all. The age of the respondent and the number of births were analysed as continuous covariates to examine their non-linear associations with caesarean section uptake.

### Statistical analysis

Cross-tabulation was used to examine the weighted percentage distribution of respondents who had a caesarean birth by the categorical covariates of the background characteristics of the respondents, using the Chi-squared test to assess statistically significant differences across the categories. Two independent sample test was used to examine the mean distribution of the continuous covariates aggregated by respondents who had a caesarean birth and those who did not. The assumption of homogeneity of variance was assessed using Levene’s test for homogeneity of variance.

To examine the extent of geographical inequalities in caesarean births at the district level, a log-binomial Bayesian Geoadditive Semiparametric (BGS) regression technique was employed [21]. The analysis was conducted for the 216 districts in Ghana [22]. The BGS approach estimates the non-linear effects of the continuous covariates and the fixed effects of the categorical and continuous covariates in addition to the unobserved spatial effects, spatially structured and unstructured [21]. The technique allows for the true underlining relationship between the outcome variable and continuous covariates to be examined. The analysis was conducted at the district level because that is where health programmes and interventions are implemented and monitored. Unique identification codes available for the geographic clusters (Primary Sampling Units) and also the individual women’s data were used to link the two data sets. In this regard, the geographic coordinates for the centroid of the geographic clusters (enumeration areas) were used to identify the respondents’ districts. See Appendix for details of the statistical methodology.

A sequential modelling approach was used to examine districts where pregnancy complications and birth risk were spatially correlated with the observed clustering of low and high caesarean births. Also, where access to health care and socioeconomic factors were associated with the observed spatial clustering. Model 0 was a null (constant) model which did not account for any covariates. Model 1 accounted for the spatial effects only. Model 2 included pregnancy complications and birth risk covariates. Model 3 added access to health care factors, whilst Model 4 included the socioeconomic factors. Only covariates significant at *p* < 0.05 were retained in the model. The statistical software R was used for the analysis [23].

## Results

### Bivariate analysis of caesarean section uptake

Table 2 shows the weighted percentage distribution of respondents who had a caesarean birth by their background characteristics. The results show that, overall, 16.0% (95% CI = 15.3, 16.8) of live births were delivered through caesarean section. A higher percentage of women who had pregnancy-induced hypertension (24.5%, 95% CI = 21.7, 27.4), prolonged/obstructed labour (26.5%, 95% CI = 22.2, 30.9), vaginal/placental (18.9%, 95% CI = 15.1, 22.6) and other complications (29.1%, 95% CI = 23.8, 34.3) delivered through caesarean section when compared to those who had no complication (13.8%, 95% CI = 12.9, 14.6), haemorrhage (12.0%, 95% CI = 9.2, 14.7) and abdominal pains (15.5%, 95% CI = 9.5, 21.4). Regarding birth risks, the results showed that a significantly (*p* < 0.001) higher percentage of births where the child was born dead, miscarried or aborted were through caesarean section when compared to those born alive. Birth weight was also significantly (*p* < 0.001) associated with caesarean births, with a higher percentage of those underweight (less than 2.5kg) and overweight (4.5 kg or higher) delivered through caesarean section.

**Table 2:** Weighted percentage distribution of respondents who had a caesarean section delivery by background characteristics

Background characteristics	% [95% CI]	n	*P*-value
Overall	16.0 [15.3, 16.8]	9244	
**Pregnancy complications and birth risks**			
Complications during and after delivery			0.000
No complication	13.8 [12.9, 14.6]	6664	
Pregnancy-induced hypertension	24.5 [21.7, 27.4]	873	
Haemorrhage	12.0 [9.2, 14.7]	481	
Prolonged/obstructed labour	26.5 [22.2, 30.9]	368	
Vaginal/placental	18.9 [15.1, 22.6]	412	
Abdominal pain	15.5 [9.5, 21.4]	179	
Other problems	29.1 [23.8, 34.3]	267	
Pregnancy outcome			0.000
Born alive	14.4 [13.6, 15.2]	7534	
Born dead	21.8 [16.5, 27.1]	219	
Miscarriage	22.8 [19.1, 26.5]	492	
Abortion	20.8 [18.7, 23.0]	999	
Birth weight of the child			0.000
Don’t know/Missing	14.0 [12.6, 15.4]	2164	
Less than 2.5 kg	21.4 [17.9, 24.9]	564	
2.5–3.99 kg	15.7 [14.7, 16.7]	5785	
4.5 kg or higher	19.9 [17.3, 22.6]	731	
**Access to health care**			
Covered by health insurance			0.000
Covered	18.1 [17.0, 19.2]	5323	
Not covered	14.0 [12.8, 15.1]	3191	
Not registered	12.5 [10.3, 14.8]	730	
Timing of first antenatal care			
First trimester	17.1 [16.2, 18.0]	6362	0.000
Second trimester	14.0 [12.7, 15.3]	2704	
After second trimester or no antenatal care	10.6 [6.5, 14.7]	178	
Place of delivery			0.197
Public facility	15.8 [15.0, 16.6]	8287	
Private facility	17.3 [15.2, 19.3]	957	
Getting permission to seek care			0.495
Big problem	15.0 [11.8, 18.1]	647	
Not a big problem	16.1 [15.3, 16.9]	8597	
Getting money needed for treatment			0.000
Big problem	13.7 [12.7, 14.8]	4509	
Not a big problem	18.0 [16.9, 19.1]	4735	
Distance, no nearby health facility			0.084
Big problem	14.8 [13.2, 16.3]	2383	
Not a big problem	16.4 [15.5, 17.2]	6861	
Not wanting to go alone			0.147
Big problem	14.4 [12.1, 16.6]	1280	
Not a big problem	16.2 [15.4, 17.0]	7964	
**Socio-economic factors**			
Type of place of residence			0.000
Urban	19.3 [18.2, 20.4]	4617	
Rural	12.0 [11.0, 13.0]	4627	
Marital status			0.000
Currently married	18.2 [17.1, 19.2]	5856	
Cohabiting	14.0 [12.7, 15.3]	2140	
Not in union	12.8 [11.1, 14.5]	1248	
Educational attainment			0.000
No education	10.6 [9.1, 12.1]	2599	
Primary	13.2 [11.4, 14.9]	1526	
Middle school/Junior High School	16.1 [14.9, 17.2]	3211	
Senior High School	19.0 [16.9, 21.1]	1219	
Higher	29.2 [25.8, 32.6]	689	
Religious affiliation			0.002
Catholic	15.8 [13.4, 18.2]	1257	
Protestant	16.4 [14.2, 18.7]	797	
Pentecostal/Charismatic	17.5 [16.3, 18.6]	3256	
Other Christians	15.4 [13.4, 17.4]	1080	
Islam	13.7 [11.9, 15.4]	2460	
No religion/Traditionalist/Spiritualist	10.6 [7.1, 14.1]	394	
Ethnicity			0.000
Akan	17.0 [15.9, 18.1]	3163	
Ga-Dangbe	16.9 [13.8, 19.9]	386	
Ewe-Guan	19.4 [17.4, 21.5]	1092	
Mole-Dagbane	10.7 [9.1, 12.2]	3149	
Grussi-Gruma-Mande	12.7 [10.4, 15.0]	1234	
Other	20.6 [15.1, 26.1]	220	
Household wealth status			0.000
Poorest	8.6 [7.1, 10.1]	2503	
Poorer	11.7 [10.2, 13.2]	1809	
Middle	13.5 [12.0, 15.1]	1704	
Rich	17.1 [15.4, 18.7]	1723	
Richest	26.3 [24.4, 28.3]	1505	
Reads newspaper			0.000
At least once a week	30.0 [25.8, 34.2]	362	
Less than once a week	19.5 [16.7, 22.3]	680	
Not at all	18.2 [16.9, 19.6]	2882	
Cannot read	12.6 [11.7, 13.6]	5320	
Listens to radio			0.000
At least once a week	17.6 [16.5, 18.7]	4407	
Less than once a week	15.8 [14.3, 17.3]	2284	
Not at all	12.6 [11.1, 14.0]	2553	
Watches television			0.000
At least once a week	18.2 [17.2, 19.2]	5031	
Less than once a week	15.1 [13.3, 16.8]	1615	
Not at all	10.2 [8.8, 11.6]	2598	

*n* Sample size

Regarding access to health care, the results show that a statistically significantly (*p* < 0.001) higher percentage (18.1%, 95% CI = 17.0, 19.2) of those covered by health insurance used caesarean section compared to those who were not covered (14.0%, 95% CI = 12.8, 15.1) or had not registered (12.5%, 95% CI =10.3, 14.8). A significantly higher percentage of those who had their first antenatal care in the first trimester had caesarean birth compared to those who had it in the second trimester or beyond and also those who did not access antenatal care. Further, those who indicated that getting the money needed for treatment was not a problem were significantly (*p* < 0.001) more likely (18.0%, 95% CI = 16.9, 19.1) to use a caesarean section, compared to those who said it was a big problem (13.7%, 95% CI = 12.7, 14.8). The differentials in the use of caesarean sections for those delivered in public and private facilities and those who indicated that it was a big problem getting permission to seek care, distance to nearby health facilities and not wanting to go alone were not significantly (*p* > 0.05) different from those who indicated otherwise.

The differentials in caesarean section use by the socioeconomic factors, type of place of residence, marital status, educational attainment, religious affiliation, ethnicity, household wealth status, reading of newspaper/literacy, listening to radio and television were all statistically significant (Table 2). The results show that a higher percentage of urban residents had caesarean section compared to their rural counterparts. A higher percentage of those married used caesarean section compared to those cohabiting or not in a union. Concerning education, an increase in educational attainment was associated with an increased percentage of caesarean section use. The results further show that a lower percentage of those with no religious affiliation, traditionalists and spiritualists delivered through caesarean section compared to Protestants and Pentecostals/Charismatics. Considering ethnicity, only the Mole-Dagbane (10.7%, 95% CI = 9.1, 12.2) and Grussi-Gruma-Mande (12.7%, 95% CI = 10.4, 15.0) ethnic groups had below 15% caesarean section rates. A marked and statistically significant (*p* < 0.001) wealth differentials exist in caesarean section uptake, with only 8.6% (95% CI = 7.1, 10.1) of women from the poorest households having caesarean birth compared to 26.3% (95% CI = 24.4, 28.3) of those from the richest households. Access to information was also associated with having a caesarean birth. The results show that a higher percentage of women who read newspapers, listen to the radio or watch television at least once a week had a caesarean birth when compared to those who did not (Table 2).

The mean of the continuous covariates, age of the respondent and the total number of births, aggregated by respondents who had a caesarean birth and those who did not are shown in Table 3. The mean age of respondents who had caesarean birth was significantly (*p* < 0.001) higher (32.4 years, 95% CI = 32.1, 32.7) compared with those who did not (30.0 years, 95% CI = 29.9, 30.2). With regards to the total number of births, the mean number of births was not statistically significantly different (p = 0.839) between those who had a caesarean birth and those who did not.

**Table 3:** Mean of the continuous covariates by respondents who had caesarean delivery and those who did not have caesarean delivery

Indicators	Mean	95% CI	*P*-value
Age of respondent in years			< 0.001
Did not have caesarean section delivery	30.0	29.9, 30.2	
Had caesarean section delivery	32.4	32.1, 32.7	
Total number of births			0.839
Did not have caesarean section delivery	2.95	2.91, 3.0	
Had caesarean section delivery	2.94	2.84, 3.0	

*CI* Confidence Intervals

### Bayesian geoadditive semiparametric analysis of geographical inequalities caesarean births

The estimated posterior prevalence ratios of caesarean section uptake for the categorical covariates and their corresponding 95% credible intervals, the variance of the spatial effects as well as their model summary statistics are shown in Table 4. The interpretation of the posterior prevalence ratios was based on the final model (Model 4) since it is the best candidate model with the lowest DIC. Model 0 was a null model which accounted for only the constant term. Model 1 added the spatial effects. When the spatial effects were included in the model, the Deviance of the null model reduced from 7530.05 to 7247.23 (a difference of 282.82). The high reduction in the Deviance when the spatial effects were included in the model shows that caesarean births in Ghana were not spatially randomly distributed but clustered. The estimated posterior variance of the structured spatial effects for Model 1 (structured spatial effect = 0.158, standard deviation = 0.067, t-statistic = 2.36) was statistically significant (*p*-value < 0.05), further confirming that there were strong spatial inequalities in caesarean births in Ghana.

**Table 4:** Posterior prevalence ratios of having caesarean delivery by the categorical covariates, their corresponding 95% credible intervals, posterior variance of the spatial effects at the district level and model summary statistics

**Background characteristics**	**Model 1**	**Model 2** **PPR [95% CI]**	**Model 3** **PPR [95% CI]**	**Model 4** **PPR [95% CI]**
**Pregnancy complications and birth risk**				
Complications during/after delivery				
No complication		1.00	1.00	1.00
Pregnancy-induced hypertension		1.84 [1.56, 2.16]**	1.81 [1.55, 2.11]**	1.78 [1.50, 2.12]**
Haemorrhage		0.87 [0.65, 1.17]	0.89 [0.67, 1.19]	0.83 [0.60, 1.13]
Prolonged/obstructed labour		1.88 [1.51, 2.35]**	1.83 [1.46, 2.30]**	1.88 [1.52, 2.33]**
Vaginal/placental		1.39 [1.11, 1.74]**	1.44 [1.16, 1.79]**	1.38 [1.04, 1.84]*
Abdominal pain		1.05 [0.68, 1.61]	1.03 [0.64, 1.65]	1.07 [0.66, 1.75]
Other problems		2.17 [1.64, 2.87]**	2.11 [1.66, 2.68]**	2.04 [1.62, 2.57]**
Pregnancy outcome				
Born alive		1.00	1.00	1.00
Born dead		1.83 [1.41, 2.37]**	1.91 [1.44, 2.53]**	1.74 [1.34, 2.26]**
Miscarriage		1.49 [1.22, 1.82]**	1.46 [1.17, 1.81]**	1.29 [1.04, 1.59]*
Abortion		1.30 [1.11, 1.52]**	1.28 [1.09, 1.50]**	1.15 [0.99, 1.33]
Birth weight of the child				
Don’t know/Missing		0.66 [0.54, 0.81]**	0.69 [0.55, 0.88]**	0.79 [0.66, 0.95]*
Less than 2.5 kg		0.98 [0.75, 1.27]	0.98 [0.77, 1.25]	1.02 [0.81, 1.30]
2.5–3.99 kg		0.70 [0.58, 0.84]**	0.69 [0.57, 0.84]**	0.72 [0.60, 0.86]**
4.5 kg or higher		1.00	1.00	1.00
**Access to health care**				
Covered by health insurance				
Covered			1.00	1.00
Not covered			0.72 [0.63, 0.82]**	0.78 [0.68, 0.89]**
Not registered			0.68 [0.54, 0.86]**	0.79 [0.62, 0.99]*
Trimester of first antenatal care				
First			1.00	
Second			0.88 [0.78, 0.99]*	
After second/no antenatal care			0.68 [0.45, 1.05]	
Getting money needed for treatment				
Big problem			1.00	
Not a big problem			1.20 [1.07, 1.35]**	
**Socio-economic factors**				
Household wealth status				
Poorest				1.00
Poorer				1.10 [0.88, 1.37]
Middle				1.27 [1.01, 1.59]
Rich				1.55 [1.26, 1.89]**
Richest				1.90 [1.51, 2.40]**
Read newspapers				
At least once a week				1.00
Less than once a week				0.73 [0.56, 0.94]*
Not at all				0.77 [0.64, 0.94]**
Cannot read				0.65 [0.51, 0.82]**
**Variance of the spatial effects**				
Structured spatial effect (SE)	0.158 (0.067)**	0.117 (0.054)**	0.131 (0.065)**	0.031 (0.020)
% change structured spatial effects	―	–25.9	+12.0	–76.3
Unstructured spatial effect (SE)	0.036 (0.021)	0.024 (0.020)	0.022 (0.015)	0.011 (0.010)
Model summary statistics				
Deviance	7247.23	7107.62	7054.01	6902.17
*P*^*D*^	51.16	52.78	55.01	54.12
$\overline{D }(\theta )$	7298.38	7160.22	7109.03	6956.29
$D(\overline{\theta })$	7247.22	7107.44	7054.02	6902.17
DIC	7349.54	7213.00	7164.04	7010.41
Change in Deviance	282.82	139.61	53.61	151.84
Change in DIC	―	136.54	48.96	153.63

Model 0 Summary statistics: Deviance = 7530.05

*PPR* Posterior Prevalence Ratio, *CI* Posterior Credible Intervals, *SE* Standard Error, *DIC* Deviance Information Criterion

^**^P<0.01; *P<0.05

**Fig. 1:**
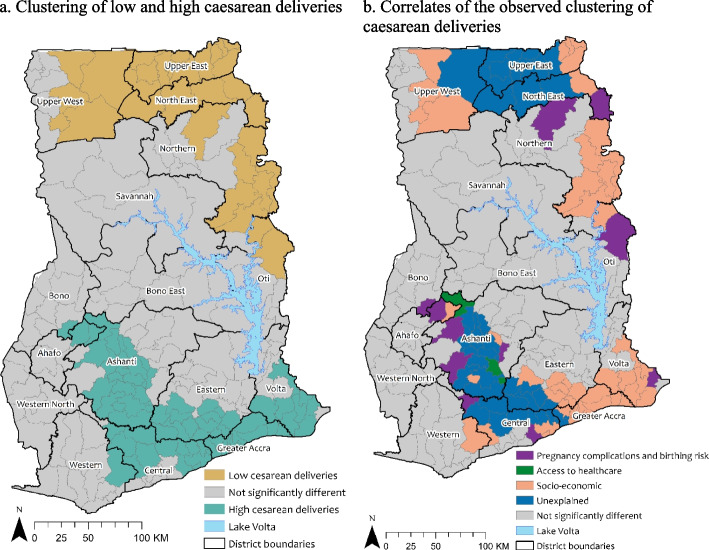
Geographical **a** clustering of caesarean deliveries in Ghana and **b** spatial covariates associated with the observed clustering

The posterior mean of the structured spatial effect from Model 1 (Fig. 1a) shows districts where without adjusting for any predictors, caesarean section uptake was statistically significantly low and also high. In all, 32 districts (14.8% of districts in Ghana) were observed to form clusters of low caesarean births, while 77 (35.6% of districts in Ghana) formed clusters of high caesarean births. There were 107 districts where the rate of caesarean births were not statistically significantly different from the average rate. These districts were observed in the middle (Savannah, Bono, Bono East and Ahafo regions) and western parts (Western and Western North regions and the coastal parts of the Central region) of the country. Parts of the Northern, Ashanti, Eastern and Volta regions were also observed as such (Fig. 1). Figure 1a further shows a clear north-south divide in the use of caesarean sections, with districts in the northern part of the country having statistically significantly low caesarean births and those in the southern part having statistically significantly high caesarean births. Caesarean births were significantly high in districts in Greater Accra, Volta, Central, Ashanti and some districts in the Eastern region. Statistically significantly low rates of caesarean births were observed among districts in the Upper West, Upper East, North East and Northern regions (Fig. 1a).

**Fig. 2:**
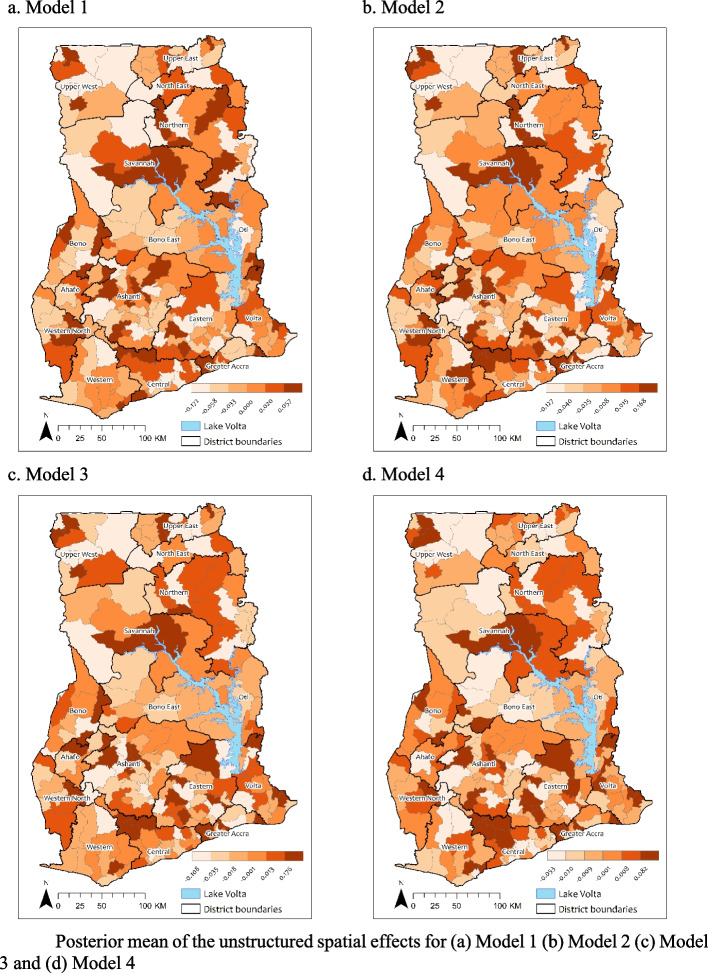
Posterior mean of the unstructured spatial effects for **a** Model 1 **b** Model 2 **c** Model 3 and **d** Model 4

Figure 2 shows the posterior mean of the unstructured spatial (random) effects. The figures show a random scatter of the posterior mean of the unstructured spatial effects for all the fitted models, indicating low spatial autocorrelation of the residuals, therefore the assumption of statistical independence and identical distribution of the residuals were not violated.

### Spatial correlates of the observed geographical clustering of caesarean section uptake

When the pregnancy complications and birth risk factors were included in the model (Table 4, Model 2), deviance was reduced by 139.61 and DIC by 136.54. Further, the posterior variance of the structured spatial effects (structured spatial effect = 0.117, standard deviation = 0.054, t-statistic = 2.17) declined by 25.9%, however, the effect remained statistically significant (*p* < 0.05). This indicates that pregnancy complications and birth risks explain some of the observed spatial clustering in caesarean births in Ghana. Figure 1b shows that pregnancy complications and birth risk factors were associated with caesarean births in eleven districts, eight of which (Gomoa West, Upper Denkyira East, Ketu North, Amansie West, Asante Akim Central Municipal, Ahafo Ano South, Tano North and Sunyani Municipal) were observed to have high caesarean births, and three (Nkwanta South, Karaga and Chereponi) with low caesarean births. The posterior prevalence ratios show that women who had pregnancy complications were significantly more likely to have a caesarean birth. Those who had pregnancy-induced hypertension, prolonged/obstructed labour and vaginal/placental complications had an increased prevalence of 1.78 (95% CI = 1.50, 2.12), 1.88 (95% CI = 1.52, 2.33) and 1.38 (95% CI = 1.04, 1.84), respectively, of having caesarean section compared with those who had no complications. The prevalence ratio of having a caesarean section was not significantly different for those who had a haemorrhage or abdominal pains when compared to those who had no complications. With regards to birth risk, the results show that caesarean section prevalence was statistically significantly higher for pregnancies where the baby was born dead (PPR = 1.74, 95% CI = 1.34, 2.26) or miscarried (PPR = 1.29, 95% CI = 1.04, 1.59) when compared to those born alive. Further, the results show that in the observed districts where pregnancy complications and birth risk were associated with caesarean births, mothers whose children’s birth weight were unknown or not reported (PPR = 0.79, 95% CI = 0.66, 0.95) and those of normal birth weight (PPR = 0.72, 95% CI = 0.60, 0.86) had reduced prevalence ratio of having caesarean birth when compared to those who were overweight (4.5 kg or higher).

When the access to health care covariates were included in the model (Model 3), the deviance and DIC reduced by 53.61 and 48.96, respectively. However, the posterior variance of the structured spatial effects (structured spatial effect = 0.131, standard deviation = 0.065, t-statistic = 2.02) increased by 12.0%, with the effect remaining statistically significant (*p* < 0.05). The increase in the posterior variance of the structured spatial effects indicates that access to health care services increases geographical inequalities in caesarean births. The results show that health insurance coverage, the timing of the first antenatal visit and access to money for treatment were significantly associated with the observed clustering of caesarean births (Table 4, Model 3). However, when the socioeconomic factors were added (Table 4, Model 4), the effect of the timing of the first antenatal visit and access to money for treatment became statistically insignificant. Thus, health insurance coverage was the only access to health care covariate that was significantly associated with the observed clustering of high caesarean section in the Bosome Freho and Offinso North districts in the Ashanti region. In these districts, women not covered and those not registered with health insurance had a reduced prevalence ratio of 22% and 21%, respectively, of having caesarean birth when compared to those who were covered by health insurance.

When the socioeconomic factors were added to the model (Model 4), the deviance and DIC declined further by 151.84 and 153.63, respectively. Furthermore, the posterior variance of the structured spatial effects (structured spatial effect = 0.031, standard deviation = 0.020, t-statistic = 1.55) declined by 76.3%, and the effect became statistically insignificant (*p* > 0.05). The large decline in the posterior variance of the structured spatial effects when the socioeconomic factors were added to the model, compared to when the pregnancy complications and birth risk (-25.9%) and also the access to health care (+12.0%) covariates were included in the model, indicates that socioeconomic covariates were the predominant factor associated with the observed clustering of caesarean section uptake in Ghana. Figure 1b shows that the socioeconomic factors were associated with high caesarean section uptake in 37 districts and low uptake in 16 districts. The results show that household wealth status and literacy (reading of newspapers) were the only socioeconomic factors statistically significantly associated with the observed clustering of caesarean section uptake. The results show that increased household wealth was associated with an increased prevalence ratio of having a caesarean birth. It is also important to note that rural-urban residence and educational attainment became statistically insignificant when household wealth status was introduced into the model. This suggests that affluence was an important motivation for caesarean section uptake in Ghana, rather than pregnancy complications and birth risks and also access to health services. Note that, the non-linear effects of the continuous covariates (age of respondent and the total number of births) had no statistically significant association with having a caesarean birth.

## Discussion

The results show a strong spatial dependency in caesarean births at the district level with a clear north-south divide. The findings show clustering of districts with significantly low rates of caesarean births in the northern part of the country and very high uptake in the southern part. Out of the 216 districts in the country, 32 were observed to form clusters of low caesarean rates, while 77 formed clusters of high caesarean rates. For the remaining 107 districts, the rate of caesarean births was not statistically significantly different from the average. With regards to the spatial correlates, the findings show that affluence (household wealth status and literacy) was the predominant associative factor of caesarean section use, rather than pregnancy complications and birth risks and also access to services. Household wealth status and literacy were associated with caesarean births in 53 districts, compared to 11 districts for pregnancy complications and birth risks and two districts for access to health care services.

Large geographical inequalities in caesarean births exist in Ghana [13]. At the district level, both overuse and underuse of caesarean sections were evident from the analysis. Underuse of caesarean section is more prevalent among districts in the northern part of the country, while overuse is prevalent among districts in the south. The northern part of Ghana is characterised by high levels of poverty, low educational advancement, poor infrastructure, and low access to services [24, 25]. On the contrary, the southern part of the country is a conglomeration of economically vibrant districts with major ports, harbours, industries and political and commercial headquarters [26]. Clearly, the low affluence of the northern part of the country and the high affluence of the southern part correlate with the observed differentials in caesarean section uptake. At the individual level, studies have reported the underuse of caesarean section among poor and less educated women and overuse among the rich and educated [17–19]. These inequalities observed at the individual level are also reflective of the geographical inequalities at the district level.

Health insurance coverage was statistically significantly associated with the observed clustering of high caesarean sections in two districts, where women covered by health insurance were more likely to have cesarean delivery. Although, Ghana’s health insurance scheme covers a range of maternity services including caesarean deliveries, studies have shown that women from wealthier households are significantly more likely to be covered when compared to those from poorer households [27]. Contrary, other studies have reported higher enrolment for very poor households [28]. Nonetheless, research evidence shows that out-of-pocket payments for maternity and allied services still exist despite fee exemptions and remain a major maternal healthcare-seeking challenge, particularly among the poor [14, 29–31]. Thus, affluence being a determinant of assessing caesarean section in Ghana as observed in this study.

This research has clearly shown that geographical inequalities in caesarean section interventions exist in Ghana and the major associative factor for seeking caesarean intervention is affluence and not pregnancy complication and birth risks or access to health services. The findings of the study call for targeted intervention such as health education to reduce overuse and unindicated caesarean sections and also policy support to promote the use of caesarean sections in underuse districts, particularly where they are medically indicated.

The limitations of the study are worth noting. The geographical location of health facilities where caesarean sections can be performed could have been used to examine the supply of the service and its impact on use, particularly in a setting like Ghana where geographical access to emergency obstetric care remains a major barrier [32, 33]. However, although there is a publicly available georeferenced list of health facilities for Ghana, there is no information on the services they provide [34]. Therefore, this data could not be used to assess the supply side of caesarean sections in the country. Nonetheless, the 2017 GMHS collected information on whether distance to a health facility was a problem or not. This information was used as a proxy for asses to health facilities.

## Conclusion

The findings of the study show that geographical inequalities in caesarean births exist in Ghana, with a strong north-south divide. Whilst caesarean births are very high in the more developed southern part of the country, they are significantly low in the poor and marginalised north. The study found that affluence is a major predictor of the inequalities observed in caesarean births among districts in Ghana. Although the World Health Organisation [3] recommends that caesarean births should only be considered when medically indicated, the results show that pregnancy complications and birth risk accounts for only a trivial amount of the observed spatial inequalities in caesarean births when compared to affluence. Although the geographical distribution of health facilities where caesarean sections were routinely performed could have explained some of the observed north-south divide, such information was not available and the 2017 GMHS data which was used for this study did not collect that information. The findings of the study suggest that target interventions and monitoring are needed to bridge the gap among districts in the use of caesarean sections.

## Data Availability

The data that support the findings of this study are publicly available upon request from The DHS Program (https://dhsprogram.com/).

## Appendix

The outcome variable of interest ${y}_{ij}$ was coded 1 if respondent *i* in district *j* had a caesarean birth and 0 otherwise. The outcome variable ${y}_{ij}$ follows a binomial distribution with the log-binomial model formulated [35] as

1 $$\mathrm{logP}({y}_{ij}=1|{\gamma }_{ij)}={\gamma }_{ij}\delta ={exp}^{{\gamma }_{ij}}\delta$$

where ${\gamma }_{ij}$ is the covariate of interest and $\delta$ are a vector of estimated regression parameters. The log link function of the log-binomial model was preferred to the logit link function of the logistic model because, unlike the logistic model, the log-binomial model has been shown to produce unbiased estimate of the adjusted prevalence ratios when the outcome of interest is not a rare (greater than 10%) event [35, 36]. If we have a vector ${x}_{ij}^\prime={({x}_{ij1},\dots ,{x}_{ijk})}^\prime$ of *k* continuous covariates and ${\lambda }_{ij}^\prime={({\lambda }_{ij1},\dots {\lambda }_{ijd})}^\prime$ a vector of *d* categorical covariates, then the predictor ${\gamma }_{ij}$ can be specified as

2 $${\gamma }_{ij}=\alpha {\lambda }_{ij}^\prime+\beta {x}_{ij}^\prime$$

Where $\delta =\alpha +\beta$, $\alpha$ is a vector of unknown regression coefficients for the categorical covariates, ${\lambda }_{ij}^\prime$,$\beta$ is a vector of unknown regression coefficients for the continuous covariates ${x}_{ij}^\prime$.

To account for the non-linear effects of the continuous covariates and the spatial correlation of the proportion of respondents who had a caesarean birth, the BGS framework, which replaces the strictly linear predictors with flexible semiparametric predictors was adopted. The model is thus specified as

3 $${\gamma }_{ij}=\alpha {\lambda }_{ij}^\prime+{f}_{k}{x}_{ijk}^\prime+{f}^{spat({S}_{i})}$$

where ${f}_{k}(x)$ are the non-linear smoothing function of the continuous variables ${x}_{ijk}$, and ${f}^{spat({S}_{i})}$ accounts for unobserved spatial heterogeneity at district *j* (*j* = 1, ..., S), some of which may be spatially structured (correlated) and others unstructured (uncorrelated). The spatially structured effects show the effect of location by assuming that geographically close areas are more similar than distant areas, whilst the unstructured spatial effect accounts for spatial randomness in the model. Equation 4 is thus specified as

4 $${\gamma }_{ij}=\alpha {\lambda }_{ij}^\prime+{f}_{k}{x}_{ijk}^\prime+{f}^{str({S}_{i})}+{f}^{unstr({S}_{i})}$$

where ${f}^{str}$ are the structured spatial effects, and ${f}^{unstr}$ are the unstructured spatial effects and ${f}^{spat({S}_{i})}={f}^{str}+{f}^{unstr}$. The spatially structured effects depict the extent of clustering in caesarean births and the associative effects of unaccounted predictor covariates, which may be spatially clustered or random. The full Bayesian approach using the Markov Chain Monte Carlo (MCMC) simulation was adopted.

The posterior mean of the structured spatial effects and their corresponding probabilities at the 95% nominal level was used to examine spatial correlates of the outcome variable at the district level. The posterior probabilities at the 95% nominal level show districts where caesarean births were statistically significantly high (high positive estimates of the posterior mean), significantly low (high negative estimates of the posterior mean), and where the effects were not significant (estimated posterior mean not significantly different from zero). The estimated posterior mean of the spatial effects characterises unexplained spatially correlated covariate information.

Model fit was examined using the Deviance Information Criterion (DIC) [37]. The DIC combines a Bayesian measure of model fit with a measure of model complexity to examine model fit [38]. The DIC is based on the posterior distribution of the deviance given by $D=-2log p\left(y|\theta \right)$, where $\left(y|\theta \right)$ is the likelihood of the observed data given the set of parameters θ. If $\overline{D }(\theta )$ is the posterior mean deviance and $D(\overline{\theta })$ is the deviance of the posterior mean, then the effective number of parameters in the model ${P}^{D}=\overline{D }\left(\theta \right)-D(\overline{\theta })$ and the $DIC=\overline{D }\left(\theta \right)+{P}^{D}$, where $\overline{D }\left(\theta \right)$ accounts for the fit of the model and ${P}^{D}$ accounts for the model complexity. Small values of DIC are associated with better models.

## Abbreviations

GMHS: Ghana Maternal Health Survey
BGS: Bayesian geo-additive semiparametric
WHO: World Health Organisation
GSS: Ghana Statistical Service
GHS: Ghana Health Service
